# Supplementary material for: The Effects of Antiperspirant Aluminum Chlorohydrate on the Development of Antibiotic Resistance in Staphylococcus epidermidis
Source: Microorganisms. 2023 Apr 5;11(4):948. doi: 10.3390/microorganisms11040948 (PMC10146609; doi:10.3390/microorganisms11040948)
Supplement: Supplementary file 1 [file microorganisms-11-00948-s001.zip › microorganisms-2266606-supplementary.pdf]

Table S1. The sequences of primers used for the Polymerase Chain Reaction.

Gene	Primer	Sequence	Tm (°C)/Amplicon (bp)	References
mecA	F	GGTCCCATTAACTCTGAAG	51/1039	[29]
	R	AGTTCTGCAGTACCGGATTTTGC		
gyrA	F	TGGCTGAATTACCTCAATCA	55/284	[26]
	R	GCCATTCTTACCATTGCTT		
gyrB	F	CAGCATTAGACGTTTCAAG	53/250	[25]
	R	CCAATACCCGTACCAAATGC		
parC	F	TCGCAATGTATTCAAGTGGG	55/197	[25]
	R	ATCGTTATCGATACTACCATT		
parE	F	AAGCTCAACAAGCACGCGAGGCTG	55/325	[25]
	R	TTAAAGTCAGTACCAACACCAGCAC		
norA	F	GCTATTATCGGTGGAGGCGTG	58/434	[27]
	R	TTTGCTTCTTTACGGCGTGAC		
norB/C	F	GCAACTAACCTTGGATGGCG	59/563	[27]
	R	ACGGTCAAGGCACTTCCGA		
16SrRNA	F	TACACACCGCCCGTCACA	55/535	[28]
	R	CTTCGACGGCTAGCTCCAAAT		
